# Hepatitis B and Delta Virus Are Prevalent but Often Subclinical Co-Infections among HIV Infected Patients in Guinea-Bissau, West Africa: A Cross-Sectional Study

**DOI:** 10.1371/journal.pone.0099971

**Published:** 2014-06-10

**Authors:** Bo Langhoff Hønge, Sanne Jespersen, Candida Medina, David da Silva Té, Zacarias José da Silva, Sharon Lewin, Lars Østergaard, Christian Erikstrup, Christian Wejse, Alex Lund Laursen, Henrik Krarup, for the Bissau HIV cohort study group

**Affiliations:** 1 Bandim Health Project, Indepth Network, Bissau, Guinea-Bissau; 2 Department of Infectious Diseases, Aarhus University Hospital, Aarhus, Denmark; 3 National HIV Programme, Ministry of Health, Bissau, Guinea-Bissau; 4 National Public Health Laboratory, Bissau, Guinea-Bissau; 5 Department of Infectious Diseases, Alfred Hospital and Monash University, Melbourne, Australia; 6 Centre for Biomedical Research, Burnet Institute, Melbourne, Australia; 7 Department of Clinical Immunology, Aarhus University Hospital, Aarhus, Denmark; 8 GloHAU, Center for Global Health, School of Public Health, Aarhus University, Aarhus, Denmark; 9 Department of Clinical Biochemistry, Aalborg University Hospital, Aalborg, Denmark; CRCL-INSERM, France

## Abstract

**Background:**

Co-infection with human immunodeficiency virus (HIV) and hepatitis B virus (HBV) may lead to accelerated hepatic disease progression with higher rates of liver cirrhosis and liver-related mortality compared with HBV mono-infection. Co or super-infection with hepatitis Delta virus (HDV) may worsen the liver disease and complicate treatment possibilities.

**Methods:**

In this cross-sectional study we included HIV-infected individuals who had a routine blood analysis performed at an HIV clinic in Bissau, Guinea-Bissau between the 28^th^ of April and 30^th^ of September 2011. All patients were interviewed, had a clinical exam performed and had a blood sample stored. The patients' samples were tested for HBV and HDV serology, and HBV/HDV viral loads were analyzed using in-house real-time PCR methods.

**Results:**

In total, 576 patients (417 HIV-1, 104 HIV-2 and 55 HIV-1/2) were included in this study. Ninety-four (16.3%) patients were HBsAg positive of whom 16 (17.0%) were HBeAg positive. In multivariable logistic regression analysis, CD4 cell count <200 cells/ µl and animist religion were significantly associated with HBsAg positivity. Due to scarcity of available plasma, virological analyses were not performed for eight patients. HBV DNA was detected in 42 of 86 samples (48.8%) positive for HBsAg and genotyping was performed in 26 patients; 25 of whom had genotype E and one genotype D. Among 9 patients on antiretroviral treatment (ART), one patient had the [L180M, M204V] mutation associated with lamivudine resistance. Among the HBsAg positive patients 25.0% were also positive for anti-HDV and 4/9 (44.4%) had detectable HDV RNA.

**Conclusion:**

HBV and HDV were frequent co-infections among HIV positive patients in Guinea-Bissau and chronic infection was associated with severe immunosuppression. Lamivudine was widely used among HBsAg positive patients with the risk of developing resistant HBV.

## Background

In West Africa, the HIV epidemic is characterized by the circulation of two distinct HIV types (HIV-1 and HIV-2). An estimated 1–2 million people are infected with HIV-2 [1] and HIV-2 is less transmissible and associated with a lower HIV RNA levels and a slower rate of CD4 cell count decline compared with HIV-1 [2]. The West African country Guinea-Bissau is currently experiencing a rise in HIV-1 prevalence and, at the same time, holds the world's highest prevalence of HIV-2 [3]. Hepatitis B is another chronic viral infection, which globally affects 350 million people and over 500.000 people die annually from hepatitis B virus (HBV)-related morbidity [4], [5]. In contrast to Europe and North America, the transmission of HBV in sub-Saharan Africa frequently occurs at birth or in early childhood [6], [7]. Infected individuals may develop cirrhosis or hepatocellular carcinoma (HCC), which is considered to be one of the most frequent causes of cancer morbidity and mortality worldwide [8]. Based on the divergence of the nucleotide sequence of viral DNA, HBV may be categorized into 8 genotypes (A–H) [9] and genotype E is most prevalent in West Africa [10]. Clinical presentation and disease progression may depend on HBV genotype and thus on geographic site of infection [11], but only few studies have been published relating genotypes to clinical outcomes in African countries [12].

The prevalence of chronic HBV (CHB) infection in people with HIV is 5–20%, with particularly high levels in many African countries [13]. In case of co-infection with HIV and HBV, the mortality rate is increased compared to HBV mono-infection with a faster rate of progression to cirrhosis and HCC [14], [15]. Furthermore, co-infected individuals have a lower chance of seroconversion towards HBV surface antigen (HBsAg), higher levels of HBV DNA [16] and an increased risk of chronicity [17].

The impact of co-infection is especially apparent in regions with widespread use of antiretroviral therapy (ART) since competing mortality from opportunistic infections is diminished. As ART becomes introduced into areas of high HBV endemicity, it is likely that liver disease from CHB will emerge as an even greater problem [18]. The nucleoside analog lamivudine and the nucleotide analog tenofovir have activity against both HIV and HBV, but when lamivudine is used as the only drug effective against HBV, viral resistance may develop [19]–[21]. Unfortunately, screening for hepatitis B co-infection is not always performed in sub-Saharan Africa due to economic shortcomings and lack of laboratory facilities [22].

Approximately 5–20% of HBsAg positive patients are co-infected with the hepatitis Delta virus (HDV), which may cause a more rapid progression of the liver disease [23], [24]. The prevalence of chronic co-infection varies with geographic region and high frequencies have been found in sub-Saharan Africa [25]. Eight genotypes of HDV have been described, termed genotype 1–8 [26]. Little information is available about HDV prevalence and impact on liver disease in sub-Saharan Africa and no previous studies have been published from Guinea-Bissau.

In this study we aimed to investigate the prevalence and clinical presentation of HBV and HBV/HDV co-infection among HIV infected patients in Guinea-Bissau.

## Methods

### Study design and sample collection

The study was conducted at the outpatient ART centre at the Hospital National Simão Mendes (HNSM) in Bissau, in collaboration with the Bandim Health Project and the National HIV Programme. The outpatient ART centre at HNSM is the largest ART centre in Guinea-Bissau and provides care for citizens of Bissau while it is also a reference center for the other HIV clinics in the country. At the first visit to the clinic, HIV testing is performed and demographic information is collected. Blood samples are usually drawn at the clinic the following day and subsequently whenever the physicians requests analysis according to national guidelines. Routines at the HIV clinic have previously been described [27].

Between April 28^th^ and 30^th^ September 2011 all HIV-positive adults who reported for routine blood analysis at the HIV clinic at HNSM were included in this study, if they provided enough blood to perform hepatitis serological analyses and HIV discriminatory testing (>0.5 ml plasma). Questionnaires about symptoms of liver disease were filled in at the day of the bleeding (nausea, vomiting, abdominal pain, skin itching, diminished libido and diminished appetite). Physical examination was performed with focus on objective signs of liver disease (icterus, dilated abdominal veins, gynaecomastia, ascites, enlarged liver, tenderness beneath right curvature, axillary alopecia and edemas) were carried out immediately after the interview.

### HIV testing

Screening for HIV was done with a rapid test in the clinic (Determine HIV-1/2 assay, Abbott laboratories, Tokyo, Japan). HIV type discrimination was performed with Genie III HIV-1/HIV-2 (Bio-Rad, Steenvorde, France) [28].

### HBV serology

Blood samples were collected in EDTA-containing tubes, centrifuged and plasma was stored at −20 degrees Celsius before transportation to the Department of Clinical Immunology, Aarhus University Hospital, Denmark where they were tested for HBsAg, anti-HBs and anti-HBc (total) by commercially available chemiluminescence assays (Architect, Abbott, Illinois, USA). The chemiluminescence assay provided signal-to-cut-off ratios (S/CO, arbitrary units), which were interpreted as “Reactive” or “Nonreactive” using pre-defined cut-offs according to the manufacturer's recommendations. Samples reactive for HBsAg were subsequently tested for HBeAg and anti-HBe. Quantitative HBsAg concentration was measured at Section of Molecular Diagnostics, Aalborg University Hospital as described elsewhere [29].

### HBV virology

HBV DNA measurements, genotyping, mutation analysis including detection of pre-core mutation were performed at Section of Molecular Diagnostics, Aalborg University Hospital, Denmark. Total DNA was isolated from 500 µl plasma using NucliSENS easyMAG Magnetic Silica (bioMérieux, Marcy l'Etoile, France) and eluted with 125 µl H_2_O. Detection and quantification of viral nucleotide sequences were performed by real-time PCR using molecular beacons on a Mx3005P Real-Time PCR System (Stratagene, La Jolla, USA). For HBV quantification, HBV-Ab and -Bb were selected from the pre-core region, amplifying a product of 189 bp. Limit of detection was 15 IU/ml (40 copies/ml). To detect pre-core mutations, sample DNA was added to each of two vials, each containing the reaction mix with 1 µmol/l of HBV 2N (wild type) or 1 µmol/l of HBV 2M (pre-core mutant). Genotype specific primer pairs and a common beacon probe from the pre-S region of the genome were used. Determination of HBV resistance was performed using direct sequencing (ABI PRISM 3130xl Genetic Analyzer, Applied Biosystems, Foster City, CA, USA) of the polymerase gene. These methods including selection of primers have been presented previously [30], [31].

### HDV analyses

Testing for anti-HDV was done using Murex anti-delta (total) (Murex Biotech Limited, Dartford, United Kingdom). A highly conserved area of the HDV genome was used to amplify a product of 175 bp in RT-PCR for HDV RNA load [32].

### Statistical analysis

We analyzed the data using χ^2^ test for categorical variables. Continuous variables were presented as means and compared using two-sample t-test (normal distribution) or as medians Wilcoxon rank-sum test (non-normal distribution). Abnormal biochemical and hematological values were defined in accordance with reference levels used at HNSM. For the analysis of risk factors of HBsAg positivity we used logistic regression and variables associated with HBsAg (p<0.10) were included in a multivariable analysis. A p-value below 0.05 was considered significant. Data was analyzed using Stata IC 11.0 (Stata Corporation, Texas, USA)

### Ethics

All patients provided a voluntary, signed and dated informed consent, or fingerprint if illiterate, prior to enrollment in the cohort. The patients consented to give blood and to the use of data provided for this study. The Danish ethical committee gave its consultative approval (case no. 1010050) and the study was finally approved by the UCEPS, the National Ethics Committee of Guinea-Bissau (N. ref. 016/CNES/2011).

## Results

### Characteristics of the included patients

Between the 28^th^ of April and the 30th of September 2011, 576 (417 HIV-1, 104 HIV-2 and 55 HIV-1/2) HIV infected patients were included (Table 1). The median age at time of inclusion was 38 years (Interquatile range (IQR): 31–47 years). Within 30 days of the interview, CD4 cell count was available from 556 (96.5%) of the patients, and the median CD4 cell count was 286 cells/ µL (IQR: 162–433 cells/ µL). Among the 302 (52.4%) patients on ART the most frequently used ART combination at inclusion was zidovudine, lamivudine and nevirapine (42.7%), followed by zidovudine, lamivudine and efavirenz (15.9%) and zidovudine, lamivudine and ritonavir boosted indinavir (13.9%).

**Table 1:** Characteristics of the 576 included HIV infected patients.

Variable	Number n/N	Percentage %
**Sex (%)**		
Male	180/574	31.4
Female	394/574	68.6
**Age stratified (%)**		
Age ≤30 years	131/549	23.9
Age 30–49 years	319/549	58.1
Age ≥50 years	99/549	18.0
**HIV-type (%)**		
HIV-1	417/576	72.4
HIV-2	104/576	18.1
HIV-1/2	55/576	9.5
**CD4 cell count (%)**		
≤200 cells/ µL	185/557	33.2
201–350 cells/ µL	158/557	28.4
>350 cells/ µL	214/557	38.4
**Nutritional status (%)**		
BMI ≤18.5 kg/m^2^	170/566	30.0
BMI >18.5 kg/m^2^	396/566	70.0
**Civil status (%)**		
Single	149/568	26.2
Married	313/568	55.1
Divorced	28/568	4.9
Widowed	78/568	13.7
**Religion (%)**		
Muslim	214/553	38.7
Catholic	174/553	31.5
Protestant	38/553	6.9
Animist	127/553	23.0
**Treatment status (%)**		
Naïve	274/576	47.6
On treatment	302/576	52.4

### HBV serology

**Figure 1:**
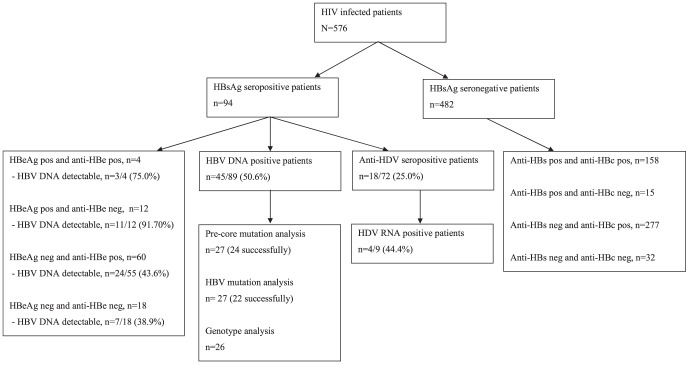
Serology and virological analyses among the HIV infected patients.

Ninety-four (16.3%) patients were HBsAg positive of whom 16 (17.0%) were HBeAg positive (Figure 1). In total 467 (81.1%) had serological markers of previous infection and 15 (2.6%) were only positive for anti-HBs. No variable from Table 1 was significantly associated with lack of serological markers of HBV. In the multivariable analysis, CD4 cell count <200 cells/ µl (odds ratio (OR) 2.11 (95% CI 1.21–3.65) p<0.01) and animist religion (OR 2.08 (95% CI 1.16–3.73) p = 0.01) were significantly associated with HBsAg positivity (Table 2). Among the HBsAg positive patients, the median CD4 cell count was lower among the HBeAg positive patients (106 cells/ µl) than the HBeAg negative patients (237 cells/ µl (p = 0.04).

**Table 2:** Risk factors for HBsAg seropositivity.

		Logistic regression, HBsAg Odds Ratio (OR)			
	Number	Univariable analysis		Multivariable analysis	
Variable	n/N (%)	OR (95% CI)	P-value	OR (95% CI)	P-value
**Sex**					
Male	180/574 (31.4)	1.22 (0.75–1.97)	0.43	-	-
Female	394/574 (68.6)	1.00	-	-	-
**Age stratified**					
Age ≤30 years	131/549 (23.9)	1.00	-	-	-
Age 30–49 years	319/549 (58.1)	1.47 (0.83–2.62)	0.19	-	-
Age ≥50 years	99/549 (18.0)	0.52 (0.21–1.25)	0.14	-	-
**HIV-type**					
HIV-1	417/576 (72.4)	1.00	-	-	-
HIV-2	104/576 (18.1)	0.60 (0.31–1.16)	0.13	-	-
HIV-1/2	55/576 (9.6)	0.48 (0.18–1.25)	0.13	-	-
**CD4 cell count**					
≤200 cells/ µL	185/556 (33.3)	2.23 (1.30–3.80)	<0.01	2.11 (1.21–3.65)	<0.01
201–350 cells/ µL	160/556 (28.8)	1.18 (0.63–2.18)	0.61	1.22 (0.65–2.28)	0.54
>350 cells/ µL	211/556 (38.0)	1.00	-	1.00	-
**Nutritional status**					
BMI ≤18.5 kg/m^2^	170/566 (30.0)	1.66 (1.03–2.66)	0.04	1.46 (0.89–2.39)	0.13
BMI >18.5 kg/m^2^	396/566 (70.0)	1.00	-	1.00	-
**Civil status**					
Married	313/568 (55.1)	1.00	-	-	-
Single	149/568 (26.2)	1.41 (0.84–2.37)	0.20	-	-
Divorced	28/568 (4.9)	1.35 (0.48–3.78)	0.57	-	-
Widowed	78/568 (13.7)	1.08 (0.54–2.17)	0.84	-	-
**Religion**					
Muslim	214/553 (38.7)	1.00	-	1.00	-
Catholic	174/553 (31.5)	1.05 (0.58–1.88)	0.88	1.06 (0.59–1.93)	0.84
Protestant	38/553 (6.9)	0.78 (0.25–2.37)	0.66	0.75 (0.24–2.32)	0.62
Animist	127/553 (23.0)	2.12 (1.20–3.77)	0.01	2.08 (1.16–3.73)	0.01
**Treatment status**					
Naïve	274/576 (47.6)	1.00	-	-	-
On treatment	302/576 (52.4)	0.87 (0.55–1.37)	0.56	-	-

### Clinical presentation of HBV infection

To evaluate the clinical presentation of HBV infected patients, we compared HBsAg positive patients with all HBsAg negative patients. HBsAg positive patients more frequently reported diminished libido (88.0% vs. 75.8% (p<0.01)) and HBsAg was associated with axillary alopecia (22.6% vs. 11.1% (p<0.01)) in the physical examination. These associations persisted after adjusting for CD4 cell count. None of the study participants were icteric, had dilated abdominal veins or male gynaecomastia.

The liver enzyme alanine transaminase (ALT) median was higher among the HBsAg positive patients (27 IU/L, IQR: 22–40 IU/L) than the HBsAg negative patients (24 IU/L, IQR: 17–31 (p<0.01)), and a higher proportion had low platelet count (14.1% vs. 6.7% (p = 0.05)). Furthermore, there was a tendency towards a higher proportion of elevated aspartate transaminase (AST) among the HBsAg positive patients (62.5% vs. 51.7% (p = 0.06)). HBsAg was not associated with serum creatinine, bilirubin, alkaline phosphatase, amylase, albumin levels or anaemia.

Among the HBsAg positive patients, HBeAg was associated with tenderness beneath right curvature (56.3% vs. 37.3%, (p = 0.02)) but no other subjective symptom or objective sign. No biochemical variable was associated with HBeAg.

### Viral characteristics of HBV infection

HBV DNA was detected in 45 (50.6%) of 89 samples positive for HBsAg (range 20 UL/ml −24×10^8^ IU/ml) and the detection rate was highest among HBeAg positive patients (Figure 1). In three cases HBV DNA was detectable but below level of quantification; all three samples were HBeAg negative and anti-HBe positive. All HBsAg positive patients on ART were receiving a regimen containing lamivudine; median duration 576 days (IQR: 316–1046 days). Fewer HBsAg positive patients on ART than without ART had detectable HBV DNA (35.7% vs. 63.8%, p<0.01). All HBsAg positive patients on ART were receiving lamivudine. Only two patients received tenofovir, and both were HBsAg negative.

Genotyping was performed successfully in 26 patients; 25 of whom had genotype E and one genotype D. The patient infected with HBV genotype D was a 33 year old woman who originated from neighboring country Guinea-Conakry. She was HIV-1/2 dually seropositive, had a CD4 cell count of 162 cells/ µl and did not receive ART at time of inclusion. All patients identified with HBV genotype E originated from Guinea-Bissau.

### HBV mutations

Twenty-seven patients were tested for pre-core mutations of whom the testing succeeded in 24 cases and eleven (45.8%) of these patients had detectable mutation. Pre-core mutation was associated with being HBeAg negative (9/11 (81.8%) vs. 3/13 (23.1%), (p<0.01)), and being anti-HBe positive (10/11 (90.9%) vs. 2/13 (15.4%), (p<0.01)). However, there was no difference in median HBV DNA levels when comparing patients with and without pre-core mutation (64×10^4^ IU/ml vs. 58×10^4^ IU/ml (p = 0.58)).

Samples from 22 patients were tested for mutations in the HBV polymerase gene (Table 3). Among 9 patients on ART, one patient had the [L180M, M204V] mutations conferring resistance to lamivudine. This patient had received 19 months of ART including lamivudine. The [V173L] mutation was found in the patient from Guinea-Conakry infected with HBV genotype D.

**Table 3:** Characteristics of 4 patients with mutations in the HBV polymerase gene.

Patient characteristics				HBV serology		HBV charateristics			
Age	Sex	HIV type	CD4 cell count	HBeAg	anti-Hbe	Genotype	Viral load	ART duration	Mutation
*Years*			*cells/ µL*				10^3^ IU/mL	*Months*	
24	F	HIV-1	91	positive	negative	E	30	19	L180M, M204V
28	F	HIV-1	436	negative	positive	E	15	-	V214A
33	F	HIV-1/2	162	positive	negative	D	40	-	V173L
66	F	HIV-1	112	negative	positive	E	66000	-	N238D

### HBsAg concentration

Quantitative HBsAg concentration was measured in all HBsAg positive patients. The median HBsAg concentration was higher among HBeAg positive patients (7128 IU/ml, IQR 1678–52068 IU/ml) than among HBeAg negative patients (3143 IU/ml, IQR 2.5–13870 IU/ml (p = 0.04)) in a Wilcoxon rank-sum test. Furthermore there was a correlation between HBV DNA levels and HBsAg concentration (Regression coefficient 4.42, 95% CI 3.52–5.33 (p<0.01)). ART status (on/without) and HBsAg concentration were not significantly correlated (median HBsAg concentration (4994 vs. 2309 IU/ml (p = 0.11)).

### HDV co-infection

Seventy-two (76.6%) of the HBsAg positive patients had provided sufficient plasma for anti-HDV testing, and 18 (25.0%) of these patients had positive serology. The distribution of HBeAg was similar among anti-HDV positive (16.7%) and anti-HDV negative patients (16.7%, p = 1.00). Variables from Table 1 were tested for association to anti-HDV, but no association was statistically significant.

Nine anti-HDV positive samples were successfully tested for HDV RNA and 4 (44.4%) samples had detectable HDV RNA ranging 82×10^2^–15×10^5^ particles/ml. One of the patients was HBeAg positive and all four were anti-HBe positive and HIV-1 infected. Only one of the HBeAg negative patients had detectable HBV DNA (23×10^2^ IU/ml) genotype E. There was no difference in the number of patients with detectable HBV DNA when comparing anti-HDV positive and negative patients (41.2% vs. 52.0%, p = 0.44).

## Discussion

In this cross-sectional study we found a high prevalence of HBV and HBV/HDV co-infections among HIV-infected patients in Guinea-Bissau. Both HBsAg and HBeAg were associated with a low CD4 cell count, but only few and relatively unspecific symptoms and objective signs were more frequent among the co-infected patients. Approximately half of the patients were on ART at time of inclusion and all HBsAg positive patients were treated with lamivudine.

Little information is available on HBV/HDV co-infections among HIV infected patients in sub-Saharan Africa and this is the first study of HBV and HBV/HDV co-infection in Guinea-Bissau. Patients were consecutively enrolled and all clinical assessments were performed by only one person limiting risk of inter-personal variability. Due to the nature of the cross-sectional design, we cannot determine the impact of HBV/HDV co-infection on morbidity and mortality on a follow-up basis. This also constitutes a risk of survival bias, as we would not have seen patients with an accelerated course of hepatitis as they would probably have died. Some patients had missing information such as no biochemistry measurements which is a limitation to the study. Due to low amounts of available plasma, complete virological analyses were not performed in all cases. Unfortunately, HDV genotypes were not determined. Only HBsAg positive patients were tested for HBV DNA and HBsAg negative patients could theoretically have an occult HBV infection underestimating the prevalence of HBV reported in this study. Missing information could cause bias in either direction of the analyses. We had limited possibilities of estimating the degree of cirrhosis, as neither liver biopsies nor fibro scan could be performed in Bissau during the study period. Finally, we did not have a control group of HIV negative patients for comparison of prevalence of HBV and HDV infections.

We found a HBV prevalence (HBsAg) among HIV infected patients of 16.3% which is in the same range as data reported from other West African countries; Cotê d'Ivore (9.0%) [33], Gambia (12.2%) [34], Burkina Faso (12.7%) [22], Ghana (13.0%) [35], Nigeria (16.7%) [36] and Senegal (16.8%) [37]. Furthermore, almost all of our patients had a serological marker of previous HBV infection. In our study HBsAg positivity was associated with a low CD4 cell count similar to some studies [36], [37] whereas other studies did not find this association [22], [34]–[35]. This association may be caused by reactivation of HBV replication after initial HBsAg seroconversion due to severe immunosuppression [38], [39], or because HBV more frequently becomes chronic among immunosuppressed patients. Thus the prevalence of HBsAg may depend on the degree of immunosuppression and HIV infected patients may have a higher HBsAg prevalence than other populations often studied e. g. blood donors and pregnant women.

The majority (68.6%) of patients in this study were females and gender imbalance was similar to the overall proportion of females at the HIV clinic (65.8%) [27]. Furthermore, males have a higher rate of loss to follow-up [27] causing the gender imbalance to enlarge during follow-up. Other sub-Saharan studies have reported a proportion of females among HIV infected patients ranging 54.8-73.6% [34]–[37].

Treating HBV/HIV co-infected patients with lamivudine have been shown to cause development of HBV resistance against this drug [19]–[21]. This could potentially be avoided if patients were given tenofovir, but during the inclusion period, this drug was only available to a few patients with severe anaemia. However, only one patient with possibly lamivudine induced resistance [L180M,M204V] mutation was found suggesting that the majority of patients had low HBV DNA levels when ART was commenced. Additionally we identified other mutations in the HBV polymerase gene of three other patients. The [V173L] mutation has also been described in a study from the nearby country Gambia. Tripple [M204V, L180M, V173L] mutation has been associated with vaccine escape mutants [20]. Although [V173L] mutations are associated with exposure to lamivudine, it has also been found in ART naïve patients previously [40]. The [V214A] and the [N238D] mutations may be considered as secondary resistance mutations against adefovir conferring very low-level resistance *in vitro* [40], however the clinical relevance of these HBV polymerase mutations in ART naïve patients still needs to be elucidated [40], [41]. Tenofovir became available for treatment of HBV/HIV co-infections in Guinea-Bissau in January 2012.

HBV genotype E has previously been documented in West Africa [10] and our study confirms the predominance of this genotype in Guinea-Bissau. In a study from Côte d'Ivore, pre-core mutations were found in 75% of the viral strains from patients negative for HBeAg and 25% of the strains from patients positive for HBeAg [42] quite similar to the data presented here. We found no difference in median HBV DNA levels when comparing patients with and without pre-core mutation. This could be due to the small sample size and the results should be interpreted with caution.

Almost all patients in this study had a serological marker of contact with HBV and fifteen patients were anti-HBs positive and anti-HBc negative; all of Guinean nationality. The latter pattern suggest previous immunization, however, childhood HBV vaccination was not introduced in Bissau until August 2008 and no vaccination campaigns have been performed among adults. Data from Greenland [30] suggest that anti-HBs-only could be a marker of HBV exposure and, thus, is not always a sign of past vaccination. Anti-HBs-only could also be due to a false positive test.

Few studies have investigated HBsAg concentration in HIV/HBV co-infected patients, and to our knowledge none of these studies have taken place in West Africa. Plasma HBsAg concentration has been suggested as a marker of therapeutic response and concentration was related to chance of anti-HBe seroconversion [43]. We found plasma HBsAg concentration to be higher among HBeAg positive patients and to correlate to HBV DNA levels. Furthermore, HBsAg concentration was independent of ART status. Additional investigations are needed to establish whether HBsAg concentration is a useful marker for treatment outcome in resource limited settings.

Large variation in the prevalence of anti-HDV among HBsAg positive pregnant women has previously been found in different regions of Senegal ranging 4.2%–44.4% [44]. Among HBsAg positive HIV infected patients in Senegal anti-HDV prevalence was 3.2% [37] and in Nigeria 2.0% of HBsAg positive patients from a gastroenterology unit tested positive for anti-HDV [45]. A recent study from Mauritania found an anti-HDV prevalence of 14.7% among HBsAg positive pregnant women and anti-HDV was associated with older age. Furthermore, the majority was identified as genotype 1 while a few were genotype 5 [46]. Another study from Mauritania also testing HBsAg positive patients from a gastroenterology unit found an even higher anti-HDV prevalence of 33.1% [47]. We found a worrying high prevalence of anti-HDV (25%), but unfortunately HDV RNA analysis was unsuccessful in a relatively large proportion of the samples due to inhibition of the PCR reaction. Treatment of HIV/HBV/HDV triple infection is complicated and ART regimens containing tenofovir seems to have limited effect on HDV replication [48].

In conclusion, we found a high prevalence of HBV and HDV co-infection in this population of HIV infected patients. Few and relatively unspecific subjective symptoms and objective signs were associated with co-infection. Although tenofovir is becoming increasingly available in Guinea-Bissau, treatment is complicated by a large proportion of HDV co-infections. Follow-up studies are necessary to determine the impact on mortality of HBV and HDV and to determine the usefulness of HBsAg concentration measurements in settings with limited resources.
