# Correction: Hepatitis B and Delta Virus Are Prevalent but Often Subclinical Co-Infections among HIV Infected Patients in Guinea-Bissau, West Africa: A Cross-Sectional Study

**DOI:** 10.1371/journal.pone.0119246

**Published:** 2015-03-03

**Authors:** The PLOS ONE Staff

The following information is missing from the Funding section: The National Cancer Institute (NCI), the Eunice Kennedy Shriver National Institute of Child Health & Human Development (NICHD) and the National Institute of Allergy and Infectious Diseases (NIAID) of the U.S. National Institutes of Health (NIH), as part of the International Epidemiologic Databases to Evaluate AIDS (IeDEA) under Award Number U01AI069919.
